# Impact of Financial Incentive for Dementia Care in Acute Care Hospitals: A Difference-in-Differences Analysis

**DOI:** 10.1093/geroni/igaf122.4262

**Published:** 2025-12-31

**Authors:** Jinyan Wu, Kojiro Morita, Ayumi Igarashi, Hideo Yasunaga, Yuya Kimura, Hiroki Matsui, Fushimi Kiyohide, Noriko Yamamoto-Mitani

**Affiliations:** Graduate School of Medicine, The University of Tokyo, Tokyo, Tokyo, Japan; Graduate School of Medicine, The University of Tokyo, Tokyo, Tokyo, Japan; Graduate School of Nursing, School of Nursing, Chiba University, Chiba, Chiba, Chiba, Japan; School of Public Health, The University of Tokyo, Tokyo, Tokyo, Japan; Graduate School of Medicine, The University of Tokyo, Tokyo, Tokyo, Japan; School of Public Health, The University of Tokyo, Tokyo, Tokyo, Tokyo, Japan; Graduate School of Medical and Dental Sciences, Institute of Science Tokyo, Tokyo, Tokyo, Japan; The University of Tokyo, Bunkyo, Tokyo, Japan

## Abstract

In 2016, Japan introduced a financial incentive motivating the establishment of multidisciplinary dementia care teams, the Dementia Care Add-on 1 (DCA1), to enhance dementia care in acute hospitals. While studies have assessed short-term effects, few have examined long-term outcomes, care process indicators, or patient-centered outcomes. To assess the long-term impact of DCA1 on older inpatients with dementia, we used data from a national inpatient database in Japan, the Diagnosis Procedure Combination, from 2014 to 2020. Difference-in-differences analysis was used to compare the outcomes between DCA1-implementing hospitals and matched controls. The primary outcome was length of stay (LOS); secondary outcomes included ADL, discharge destination, in-hospital fractures, and potentially inappropriate medication prescriptions. Among 235 matched hospital pairs (309,791 patients), DCA1 implementation alone was not significantly associated with outcomes. Sensitivity analysis showed patients who received care billed under DCA1 (41.5% of eligible cases), were more likely to have reduced LOS (difference, −1.57 days; 95% confidence interval (CI), −2.09 to − 1.06), maintained ADL (odds ratio, 1.96; 95%CI, 1.23 to 3.12) and to have been discharged home (odds ratio, 2.03; 95%CI, 1.28 to 3.20). Another sensitivity analysis excluding hospitals certified for DCA2 (a less stringent version of the incentive) showed modest LOS reduction (difference, –0.12 days; 95%CI, −0.22 to − 0.03). Over 60% of DCA1 hospitals billed fewer than 20% of eligible patients, indicating limited implementation. These findings suggest that the policy itself may have a limited overall impact; however, its effectiveness depends on actual care delivery. Future policies should prioritize implementation quality and contextual support.

